# Altered Dynamics of *Candida albicans* Phagocytosis by Macrophages and PMNs When Both Phagocyte Subsets Are Present

**DOI:** 10.1128/mBio.00810-13

**Published:** 2013-10-29

**Authors:** Fiona M. Rudkin, Judith M. Bain, Catriona Walls, Leanne E. Lewis, Neil A. R. Gow, Lars P. Erwig

**Affiliations:** Aberdeen Fungal Group, Institute of Medical Sciences, University of Aberdeen, Aberdeen, United Kingdom

## Abstract

An important first line of defense against *Candida albicans* infections is the killing of fungal cells by professional phagocytes of the innate immune system, such as polymorphonuclear cells (PMNs) and macrophages. In this study, we employed live-cell video microscopy coupled with dynamic image analysis tools to provide insights into the complexity of *C. albicans* phagocytosis when macrophages and PMNs were incubated with *C. albicans* alone and when both phagocyte subsets were present. When *C. albicans* cells were incubated with only one phagocyte subtype, PMNs had a lower overall phagocytic capacity than macrophages, despite engulfing fungal cells at a higher rate once fungal cells were bound to the phagocyte surface. PMNs were more susceptible to *C. albicans*-mediated killing than macrophages, irrespective of the number of *C. albicans* cells ingested. In contrast, when both phagocyte subsets were studied in coculture, the two cell types phagocytosed and cleared *C. albicans* at equal rates and were equally susceptible to killing by the fungus. The increase in macrophage susceptibility to *C. albicans*-mediated killing was a consequence of macrophages taking up a higher proportion of hyphal cells under these conditions. In the presence of both PMNs and macrophages, *C. albicans* yeast cells were predominantly cleared by PMNs, which migrated at a greater speed toward fungal cells and engulfed bound cells more rapidly. These observations demonstrate that the phagocytosis of fungal pathogens depends on, and is modified by, the specific phagocyte subsets present at the site of infection.

## IMPORTANCE

Extensive work investigating fungal cell phagocytosis by macrophages and PMNs of the innate immune system has been carried out. These studies have been informative but have examined this phenomenon only when one phagocyte subset is present. The current study employed live-cell video microscopy to break down *C. albicans* phagocytosis into its component parts and examine the effect of a single phagocyte subset, versus a mixed phagocyte population, on these individual stages. Through this approach, we identified that the rate of fungal cell engulfment and rate of phagocyte killing altered significantly when both macrophages and PMNs were incubated in coculture with *C. albicans* compared to the rate of either phagocyte subset incubated alone with the fungus. This research highlights the significance of studying pathogen-host cell interactions with a combination of phagocytes in order to gain a greater understanding of the interactions that occur between cells of the host immune system in response to fungal invasion.

## INTRODUCTION

*Candida* species collectively represent the fourth leading cause of nosocomial bloodstream infections in the United States (1, 2). Within the *Candida* genus, *Candida albicans* is the major human fungal pathogen, accounting for approximately 50% of candidemia cases (3, 4). This fungus ordinarily colonizes mucosal surfaces of the skin and gastrointestinal and vulvovaginal tracts of healthy individuals. Around 75 million women suffer at least four episodes of *Candida* vaginitis each year, and AIDS-associated esophagitis is extremely common in untreated HIV-positive individuals (3). This opportunistic pathogen can also cause life-threatening systemic infections, with associated mortality rates of 30 to 60% in patients who have implanted medical devices, have been administered immunosuppressant drugs, or have been subjected to severe trauma (5).

An important first line of defense against *Candida* infections is provided by the innate immune system through the recruitment of professional phagocytes, such as polymorphonuclear cells (PMNs) and macrophages, to the site of infection (5–8). Studies to date have recognized the importance of macrophages and neutrophils in host defense against fungal pathogens and suggest a dominant role for the PMNs (9–11). This is reflected in the observation that neutropenic patients and patients with peroxidase deficiency are highly susceptible to invasive candidiasis (5, 12, 13). In particular, patients with chronic granulomatous disease, displaying impaired neutrophil function, are especially prone to systemic fungal infections (12–15). Additionally, in a mouse model where splenic macrophages were eliminated, mice demonstrated increased susceptibility to experimental disseminated candidiasis (16). Despite studies highlighting the clinical importance of phagocytes in anti-*Candida* infection, the process of fungal cell phagocytosis remains poorly understood at the mechanistic level.

The first step in the phagocytosis process involves macrophages and PMNs recognizing pathogen-associated molecular patterns (PAMPs) present in the fungal cell wall through pattern recognition receptors (PRRs) localized on the phagocytic cell membrane, in endosomes, and in the cytoplasm (17–19). Engagement of these receptors enables the phagocyte to directly engulf and destroy fungal cells within the phagolysosome by a number of oxidative and nonoxidative mechanisms, including the production of toxic reactive oxygen species (ROS) and reactive nitrogen species (RNS), expression of various antimicrobial peptides, and the activities of hydrolytic enzymes (5, 6, 17, 20–23). Furthermore, fungi and bacteria induce the formation of neutrophil extracellular traps (NETs) by activated PMNs, which can entrap and kill fungi and bacteria extracellularly (24). PAMP-PRR interactions also facilitate indirect killing of *Candida* by triggering the induction of proinflammatory cytokines and chemotactic factors which serve to activate other arms of the host immune system and aid in the clearance of *Candida* from the body (6, 17, 18).

Extensive work has been carried out identifying the PRRs and downstream signaling pathways that are involved in phagocyte recognition of fungal cells (6, 9, 23). These studies have identified the Toll-like receptors (TLRs) and C-type lectin receptors (CLRs) as the major PRRs involved in *C. albicans* PAMP recognition (6, 17, 25). Other studies have revealed how the overall phagocytic process in macrophages and PMNs is affected by fungal cell wall composition, morphogenesis, and species (5, 8, 26, 27). For example, Keppler-Ross et al. demonstrated that J774.1 macrophages preferentially phagocytosed *Candida glabrata* and *Saccharomyces cerevisiae* over *C. albicans* and that these macrophages displayed a strong preference for *C. albicans* yeast cells rather than hyphal cells (26). In addition, *C. albicans* mutants deficient in cell wall *N*- and *O*-linked glycans negatively affected the capacity of PMNs to phagocytose *C. albicans* (5). Such studies have been informative but have investigated phagocytosis under conditions where only one immune cell type and one fungal cell type were present.

By breaking down the phagocytic process into distinct stages (migration, recognition, engulfment, phagosome maturation, and killing), we have been able to determine the effect of specific factors, such as cell morphology and cell wall composition, on individual stages of the phagocytosis process (8, 28). In doing so, novel insights into the mechanisms that govern effective phagocytic clearance have been revealed. For example, dynamic imaging was used to demonstrate nonlytic expulsion of hyphal *C. albicans* cells from macrophages (29) and *C. albicans*-mediated inhibition of cell division in macrophages undergoing mitosis (30).

In this study, we assessed stage-specific *C. albicans* phagocytosis by human monocyte-derived macrophages and human PMNs in isolation with *C. albicans* and when both phagocyte subsets were present. Through this dual approach, we revealed marked differences in the behaviors of cells and in the outcomes of the interaction between PMNs and macrophages with *C. albicans* during phagocytosis. We show a lower overall capacity of PMNs to phagocytose fungal cells, an increased susceptibility of PMNs to *C. albicans*-mediated killing when phagocytes were incubated alone with *C. albicans*, and a marked increase in *C. albicans*-mediated macrophage killing when both types of phagocytes were cocultured with *C. albicans*.

## RESULTS

### Comparison of macrophage- and PMN-mediated *C. albicans* phagocytoses.

**FIG 1:**
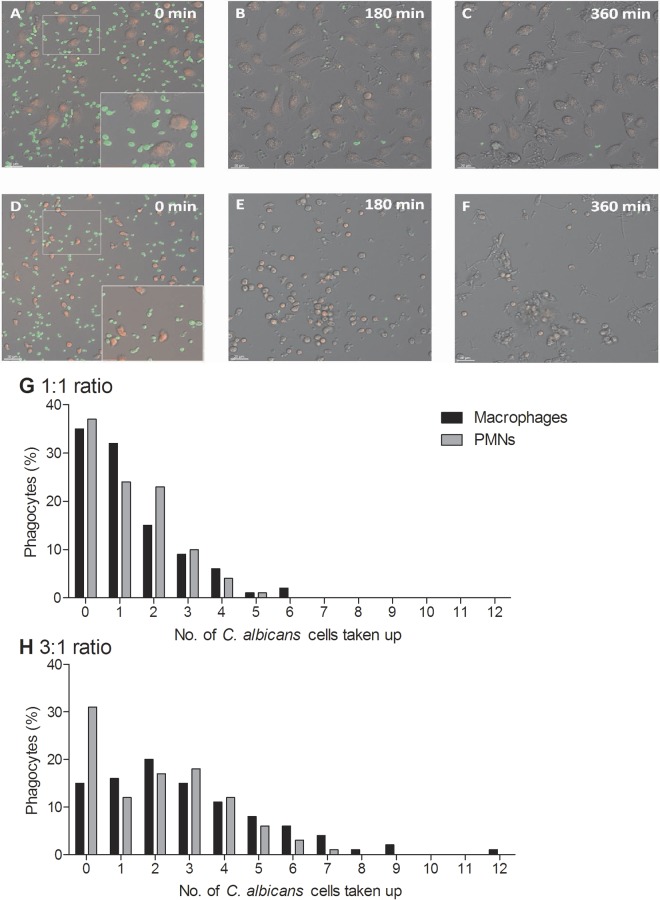
Uptake of live *C. albicans* cells by macrophages and PMNs. (A to F) Snapshots from live-cell video microscopy experiments tracking the phagocytosis of live wild-type *C. albicans* cells by human monocyte-derived macrophages (A to C) and human PMNs (D to F) over 6 h. Phagocytes (red) with live wild-type *C. albicans* cells (green) at 0 min, 180 min, and the end of the 6 h. Bars, 30 µm. (G and H) Numbers of *C. albicans* cells ingested by macrophages and PMNs at the 1:1 (G) and 3:1 (H) *C. albicans* cell/phagocyte ratios. Bars represent percentages of macrophages or PMNs that ingested a defined number of *C. albicans* cells.

Phagocytosis of *C. albicans* by macrophages and PMNs is critical for elimination of fungal infections from the body; however, the relative roles that they play in this process and the interactions that occur between these two cell types remain to be elucidated. We first quantified the dynamics of *C. albicans* phagocytosis by PMNs or macrophages in cell cultures using pure populations of only one phagocyte type. To do this, human monocyte-derived macrophages and human PMNs were challenged with live *C. albicans* CAI4-CIp10. Live-cell video microscopy using our standard phagocytosis assay (28) recorded the total number of *C. albicans* cells taken up by individual macrophages and PMNs over a 6-h period. Video S1 in the supplemental material shows the interactions between *C. albicans* and macrophages, and Videos S2 to S4 show the interactions between *C. albicans* and PMNs. An uptake event was defined as the complete engulfment of one *C. albicans* cell by either a macrophage or a PMN following cell-cell contact. Figure 1A to C and D to F show snapshots of the overall clearance of *C. albicans* cells throughout the 6 h of the assay by macrophages and PMNs, respectively. At a multiplicity of infection (MOI) of 1, there was no significant difference between the macrophages and PMNs in terms of the average numbers of *C. albicans* cells taken up (Table 1) or the percentages of cells taking up at least one fungal cell (65.0 ± 3.6 and 62.7 ± 10.4, means ± standard deviations [SD] [*n* = 300], for macrophages and PMNs, respectively) (Fig. 1G). However, when the MOI was increased to 3, the average *C. albicans* cell uptake by macrophages was significantly greater than for PMNs (Table 1) (*P* < 0.05). Furthermore, the percentage of macrophages taking up at least one fungal cell was significantly greater than for PMNs (85.0 ± 3.6 and 68.7 ± 4.0, respectively) (Fig. 1H). Thus, at low concentrations of *C. albicans* cells, both phagocytes were equally as effective in taking up fungal cells; however, when *C. albicans* yeast cells were in excess, macrophages took up a greater number of *C. albicans* cells than PMNs did.

**TABLE 1:** Uptake of live *C. albicans* cells by macrophages and PMNs^*a*^

Phagocytes	Avg uptake (no. of *C. albicans* cells taken up ± SD)		
	*C. albicans* cell/phagocyte MOI of 1	*C. albicans* cell/phagocyte MOI of 3	Coculture assay
Macrophages	1.3 ± 0.2	2.9 ± 0.3	2.3 ± 0.6
PMNs	1.2 ± 0.1	2.0 ± 0.3*	2.5 ± 0.7

^a^ Average number of *C. albicans* cells taken up ± SD by human monocyte-derived macrophages and human PMNs. *n* = 3; *, *P* < 0.05.

### Human PMNs engulf *C. albicans* more rapidly than macrophages do.

**FIG 2:**
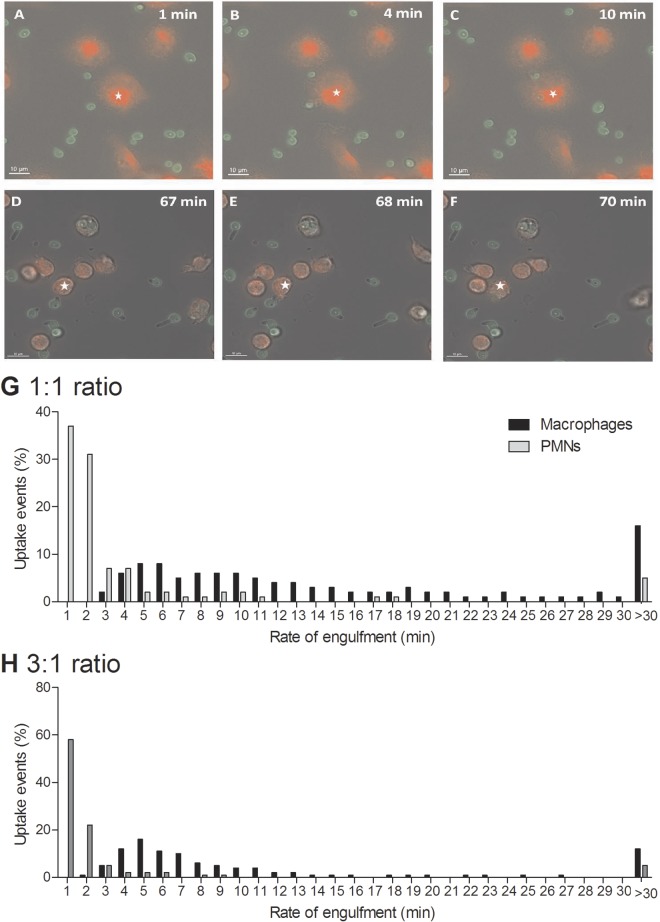
Macrophage and PMN engulfment of live *C. albicans* cells. Shown are snapshots taken from live-cell video microscopy capturing the various stages of *C. albicans* engulfment by macrophages (A to C) and PMNs (D to F). (A and D) Phagocyte (red, ☆) and *C. albicans* cell (green) prior to cell-cell contact; (B and E) cells once cell-cell contact had been established; (C and F) *C. albicans* within the phagocyte following ingestion; (G and H) times taken for macrophages and PMNs to ingest live wild-type *C. albicans* cells at the 1:1 (G) and 3:1 (H) *C. albicans* cell/phagocyte ratios. The rate of engulfment was defined as the time taken from initial cell-cell contact to complete ingestion of *C. albicans* cells by the phagocyte. Bars represent the percentages of uptake events (*n* = 3).

Uptake of target yeast cells is influenced by the migration of phagocytes toward the site at which fungal cells are located and the time taken from the establishment of cell-cell contact to complete uptake of *C. albicans*. As in our previous studies, we define the latter as the rate of engulfment (28). We examined whether there were differences in the rates of *C. albicans* engulfment between macrophages and PMNs by live-cell video microscopy and image analysis (Fig. 2A to C and D to F for macrophages and PMNs, respectively). First, at MOIs of both 1 and 3, PMNs engulfed *C. albicans* cells at a significantly higher rate than macrophages (Table 2; Fig. 2G and H). Videos S1 to S3 in the supplemental material highlight this observed difference in rates of engulfment between macrophages and PMNs. Furthermore, at both MOIs, approximately 12 to 15% of *C. albicans* cells bound to a macrophage took longer than 30 min to be internalized, compared with 5% of cells bound to PMNs (Fig. 2G and H). Interestingly, when macrophage activities at the different MOIs were compared, it was observed that macrophages engulfed *C. albicans* cells at a markedly higher rate at a MOI of 3 than at an MOI of 1 (Table 2). This accelerated uptake of yeast cells did not occur for PMNs at higher MOIs (Table 2).

**TABLE 2:** Rate of engulfment of live *C. albicans* cells by macrophages and PMNs^*a*^

Phagocytes	Avg rate of engulfment (min ± SD)		
	*C. albicans* cell/phagocyte MOI of 1	*C. albicans* cell/phagocyte MOI of 3	Coculture assay
Macrophages	12.0 ± 1.3	8.1 ± 0.4^*b*^	14.4 ± 0.6^*c*^
PMNs	2.9 ± 0.6^*d*^	2.1 ± 0.4^*d*^	1.7 ± 0.2^*d*^

^a^ Average times (min) taken ± SD for human monocyte-derived macrophages and human PMNs to engulf live wild-type *C. albicans* cells (*n* = 3).

^b^ The value for macrophages was significantly different at an MOI of 3 (*P* < 0.01) from the value at an MOI of 1 when macrophages were incubated alone with *C. albicans*.

^c^ The value for macrophages in coculture was significantly different (*P* < 0.0001) from that for macrophages incubated alone at an MOI of 3.

^d^ Values for PMNs were significantly different (*P* < 0.0001) from those for macrophages at the same MOI.

In summary, PMNs engulfed *C. albicans* at a significantly higher rate than macrophages did at both a low and a high MOI, and the rate of macrophage engulfment was affected by the concentration of fungal cells.

### PMNs are more susceptible than macrophages to killing by *C. albicans*.

**FIG 3:**
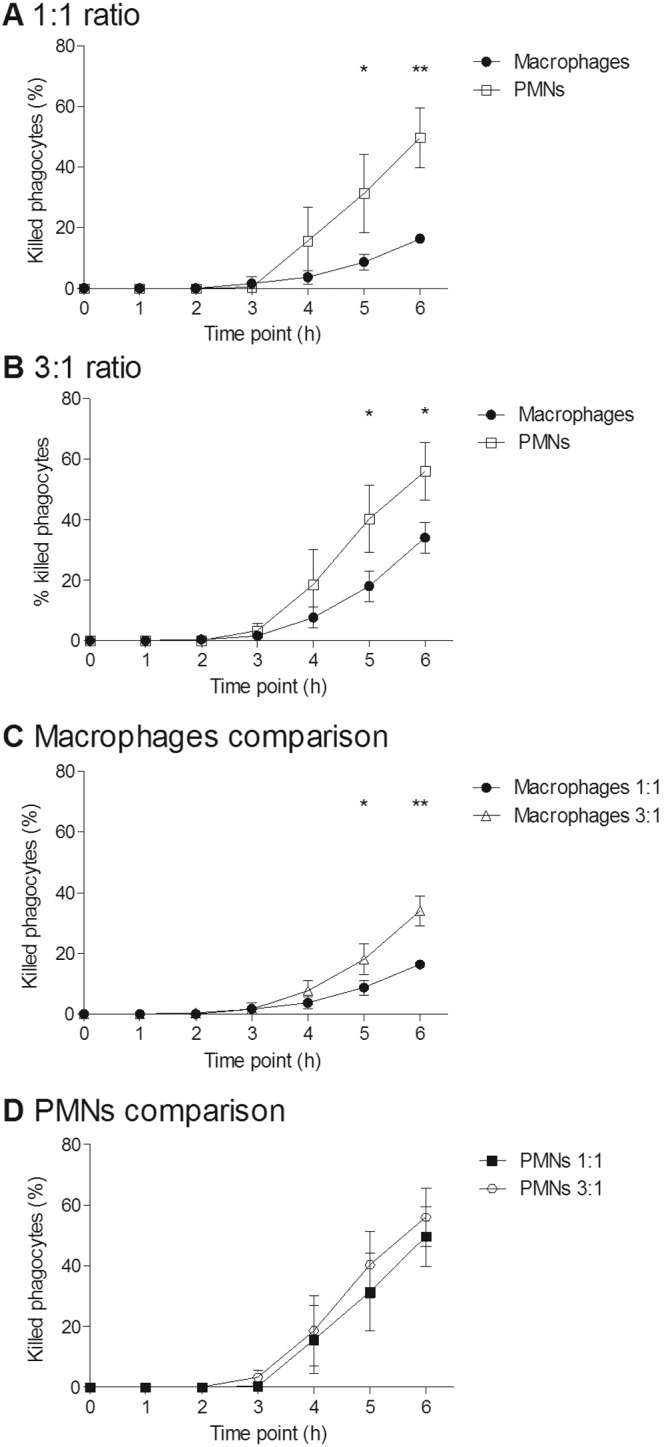
Killing of macrophages and PMNs by live *C. albicans* cells over a 6-h period. (A and B) Percentages of macrophages and PMNs killed by *C. albicans* at the 1:1 (A) and 3:1 (B) *C. albicans* cell/phagocyte ratios; (C and D) comparisons of *C. albicans*-mediated phagocyte killings at MOIs for macrophages (C) and PMNs (D). Values are means ± SD (*n* = 3). *, *P* < 0.05; **, *P* < 0.01.

**FIG 4:**
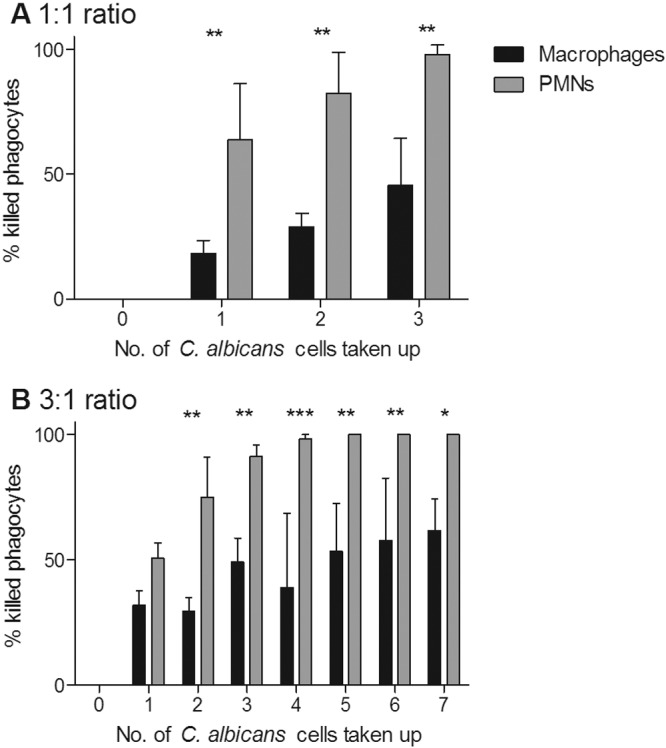
*C. albicans*-mediated phagocyte killing in relation to the number of *C. albicans* cells taken up. (A and B) Percentages of phagocytes killed after taking up a defined number of *C. albicans* cells at the 1:1 (A) and 3:1 (B) *C. albicans* cell/phagocyte ratios. Bars represent mean percentages of phagocytes killed ± SD (*n* = 3). *, *P* < 0.05; **, *P* < 0.01; ***, *P* < 0.001.

It has been shown previously that *C. albicans* can form hyphae inside phagocytes, often resulting in phagocyte rupture and death (8, 31, 32). We examined this phenomenon in more detail to determine whether there were differences between the different phagocytic cell types in this respect. It was observed that at both low and high MOIs, a significantly greater number of PMNs than macrophages were killed by *C. albicans* during the course of the phagocytosis assay (Fig. 3A and B). As expected, at the higher MOI, significantly more macrophages were killed than at the lower MOI (Fig. 3C). In contrast, rates of PMN killing were similar at both MOIs (Fig. 3D). The percentages of macrophages and PMNs killed increased with the number of fungal cells being taken up; however, it was observed that at both MOIs, when macrophages and PMNs had taken up the same number of *C. albicans* cells, a significantly greater proportion of PMNs were killed (Fig. 4A and B). This may reflect the smaller average size of PMNs (8.3 µm ± 1.1 µm) than macrophages (21.2 µm ± 3.5 µm). Furthermore, the data revealed that >90% of PMNs that took up 3 or more fungal cells were killed, whereas a high proportion (~40%) of macrophages survived even after ingesting up to 7 fungal cells. Thus, in these assays, PMNs were more susceptible to *C. albicans*-mediated killing than macrophages, irrespective of the number of *C. albicans* cells ingested.

### Phagocytosis and killing dynamics in mixed-phagocyte cocultures.

For coculture assays, an MOI of 3 *C. albicans* cells for all phagocytes (macrophages plus PMNs, with macrophages and PMNs at a 1:1 ratio) was used. *C. albicans* uptake and engulfment by macrophages and PMNs was quantified, and *C. albicans*-mediated phagocyte killing was measured (see Video S5 in the supplemental material).

**FIG 5:**
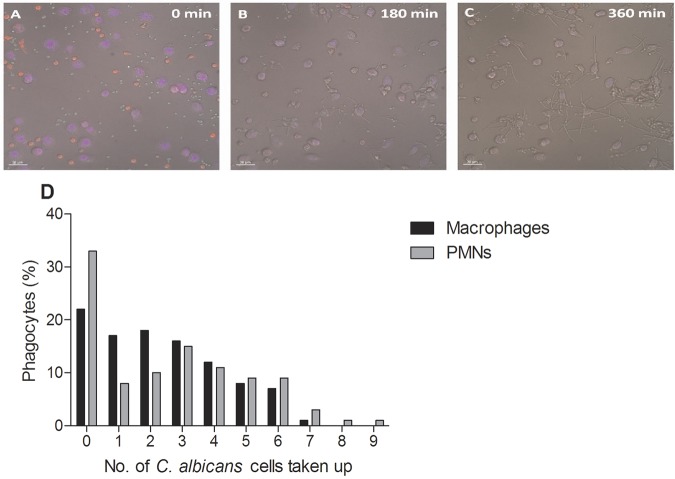
Uptake of live *C. albicans* cells in combined macrophage-PMN cultures. (A to C) Images taken from live-cell video microscopy experiments tracking the phagocytosis of live *C. albicans* cells by macrophages and PMNs in the combined assay over 6 h. Shown are macrophages (blue) and PMNs (red) with live *C. albicans* cells (green) at 0 min, 180 min, and the end of the 6 h. Bar, 30 µm. (D) Numbers of *C. albicans* cells ingested by macrophages and PMNs in the combined assay. Experiments were carried out an MOI of 3. Bars represent percentages of macrophages or PMNs that ingested a defined number of *C. albicans* cells (*n* = 3).

When phagocytes were cultivated with an excess of *C. albicans* cells, macrophages took up more fungal cells than PMNs did. We examined whether the same was true when macrophages and PMNs were present in coculture. Interestingly, no significant difference between macrophages and PMNs was observed in *C. albicans* uptake rates (Table 1 and Fig. 5A to C). Additionally, approximately 22% of PMNs did not take up any *C. albicans* cells, compared with 33% of macrophages (Fig. 5D). Thus, when both cell types were present, macrophages and PMNs contributed approximately equally to clearance of *C. albicans*.

### Human PMNs engulf *C. albicans* more rapidly than macrophages do in mixed-phagocyte cocultures.

**FIG 6:**
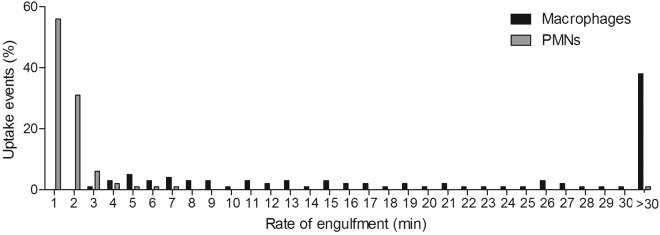
Macrophage and PMN engulfment of live *C. albicans* cells in combined cultures. Shown are the times taken for macrophages and PMNs to ingest live *C. albicans* cells in the coculture assay. Rate of engulfment was defined as the time taken from initial cell-cell contact to complete ingestion of *C. albicans* by the phagocyte. Experiments were carried out at a MOI of 3. Macrophages and PMNS were present at a 1:1 ratio. Bars represent the percentages of uptake events (*n* = 3).

When both phagocyte subsets were present, PMNs engulfed *C. albicans* at a significantly higher rate than macrophages (Table 2 and see Video S5 in the supplemental material). Furthermore, 38% of *C. albicans* cells took longer than 30 min to be engulfed by macrophages, whereas only 1% of fungal cells took longer than 30 min to be ingested by PMNs (Fig. 6). The average rate of *C. albicans* engulfment by PMNs was similar to that observed when the PMNs were studied in isolation (Table 2 and Fig. 2H and 6). In contrast, macrophage engulfment of *C. albicans* was significantly delayed in the coculture assay, with macrophages taking an average of 14.4 ± 0.6 min to engulf fungal cells, compared to 8.1 ± 0.4 min in the absence of PMNs (Table 2 and Fig. 2H and 6). Thus, the rate of *C. albicans* engulfment by macrophages decreased when PMNs were present, whereas for the PMNs, the rate of engulfment was unaffected by the presence of macrophages and was significantly higher both in the presence and in the absence of macrophages.

### *C. albicans*-mediated macrophage killing increases in mixed-phagocyte cocultures.

**FIG 7:**
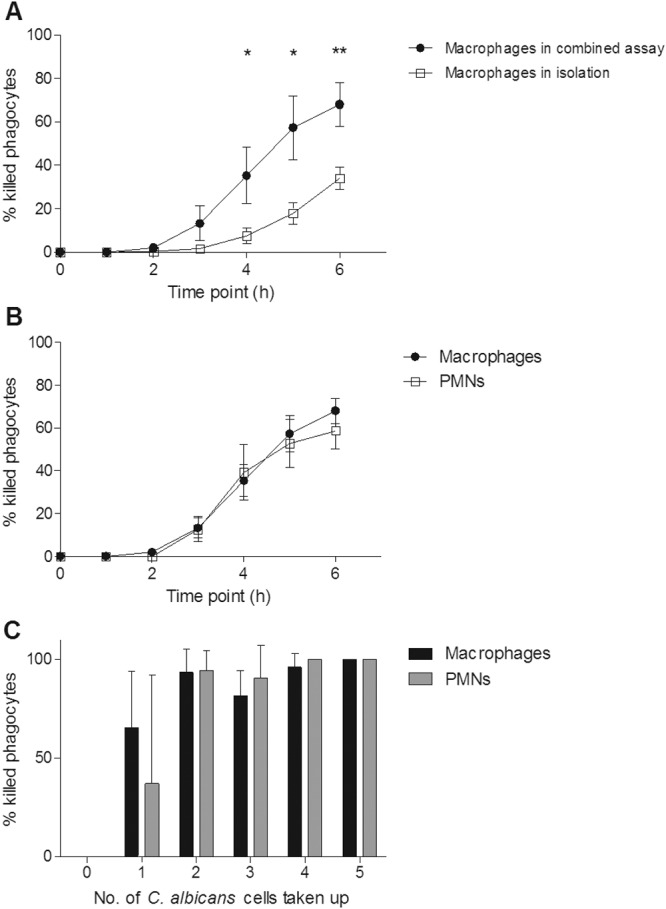
Killing of macrophages and PMNs by live *C. albicans* cells in the combined assay over 6 h. (A) Comparison of *C. albicans*-mediated killing of macrophages in the combined assay and that in isolation; (B) percentages of macrophages and PMNs killed by *C. albicans* in the combined culture assay; (C) percentages of phagocytes killed after taking up a defined number of *C. albicans* cells in the combined assay. Experiments were carried out at a 3:1 ratio of *C. albicans* cell to total phagocytes. Values/bars represent means ± SD (*n* = 3). *, *P* < 0.05; **, *P* < 0.01.

In the coculture assay, *C. albicans*-mediated macrophage killing was significantly increased from the level observed when macrophages were cultivated in isolation with *C. albicans* (Fig. 7A). Indeed, there was no difference in rates of *C. albicans*-mediated killing of macrophages and PMNs when both cell types were present (Fig. 7B). This contrasts with the observation that PMNs were significantly more susceptible to killing by fungal cells when PMNs and macrophages were cultured in isolation (Fig. 3B). Additionally, the percentage of macrophages killed after taking up 2 or more fungal cells in the coculture assay was >80%, compared to approximately 25% when macrophages were incubated alone with *C. albicans* (Fig. 4B and 7C).

### PMNs migrate more rapidly toward *C. albicans* cells.

**FIG 8:**
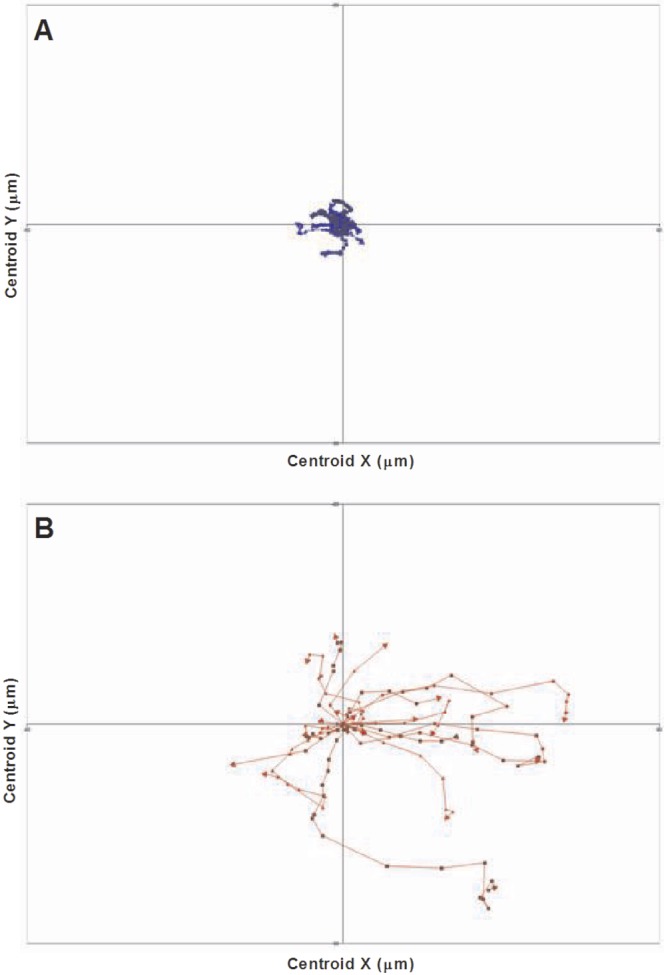
Phagocyte migration toward *C. albicans*. Shown are tracking diagrams illustrating the distances traveled, directionality, and velocity of macrophages (A, blue) and PMNs (B, red) in pursuit of live *C. albicans* cells in the combined assay. Tracks represent the movement of individual phagocytes relative to their starting position, symbols indicate the locations of phagocytes at 1-min intervals, and arrows represent directionality.

To investigate this increase in *C. albicans*-mediated macrophage killing, we analyzed the migration of macrophages and PMNs toward fungal cells. *C. albicans* cells formed germ tubes rapidly under the conditions of the experiment; therefore, the time taken for a phagocyte to locate and migrate toward a fungal cell influenced whether it was taken up early in the yeast form or later on as a germ tube. It is reasonable to speculate that this may ultimately affect the susceptibility of the phagocyte to *C. albicans*-mediated killing. Tracking diagrams (Fig. 8A and B) illustrate the distances traveled and the directionality and velocity of PMNs and macrophages in the coculture assay in the presence of wild-type *C. albicans*. In these diagrams, symbols indicate the locations of macrophages or PMNs at 1-min intervals, and arrows represent directionality. Quantitative analysis of the average track revealed a 20-fold-higher average track velocity of PMNs than of macrophages toward *C. albicans* cells (0.007 ± 0.004 µm/s [*n* = 3] for macrophages versus 0.13 ± 0.07 µm/s [*n* = 3] for PMNs) (see Video S5 in the supplemental material). Thus, in the combined phagocyte assays, PMNs migrated at a greater velocity than macrophages toward *C. albicans*.

This faster migration of PMNs toward fungal cells resulted in earlier clearance of yeast cells by PMNs, leaving the remaining *Candida* cells time to germinate before being engulfed by macrophages as hyphae. Indeed, in the coculture assays, macrophages took up a significantly greater proportion of hyphal cells (relative to yeast cells) in the coculture assay than they did when in isolation (72.4% ± 9.6% and 59.3% ± 2.7% [means ± SD, *n* = 300] of hyphal cells were taken up by macrophages in coculture and isolation assays, respectively [data not shown]), and this coincided with increased *C. albicans*-mediated macrophage killing. These data are consistent with previous reports which indicate that uptake of hyphal cells enhances phagocyte killing (8). In addition, this increase in hyphal uptake was accompanied by a lower rate of engulfment than when macrophages were incubated alone with *C. albicans* (Table 2). Furthermore, in some uptake events, we observed continued extension of *C. albicans* hyphae during the engulfment process (see Video S6 in the supplemental material). These are all factors which are likely to have contributed to enhanced macrophage killing. Thus, in the presence of PMNs, macrophages were more susceptible to killing by the fungus.

## DISCUSSION

*C. albicans* is a major life-threatening human fungal pathogen that causes infections associated with high morbidity and high mortality (2). Professional phagocytes, such as macrophages and PMNs, provide an important line of defense against invasive candidiasis by directly engulfing and destroying fungal cells. We conducted a detailed comparison of stage-specific phagocytosis of *C. albicans* by PMNs and that by macrophages in isolation and when both phagocyte subsets were present and competing for fungal cells. We observed profound differences in (i) migration, (ii) rate of engulfment, (iii) overall uptake, and (iv) phagocyte survival when *C. albicans* yeast cells interacted with mixtures of macrophages and PMNs rather than individual phagocyte subsets. Nonprofessional phagocytes, such as dendritic and endothelial cells, also contributed to the overall clearance of *C. albicans in vivo* and will be the subject of future studies*.*

PMNs are considered the primary mediators of phagocytic clearance (9–11, 31, 33). Indeed, neutropenia is a major predisposing factor in clinical studies for developing invasive candidiasis (21, 22). Furthermore, recent *in vitro* studies suggest that the onset of hyphal formation and subsequent escape of fungal cells is inhibited inside human neutrophils (33). This may suggest that PMNs not only mediate the majority of *C. albicans* uptake but also are less susceptible than macrophages to killing by *C. albicans* following phagocytosis. Surprisingly, we observed that when only one immune cell type was present, macrophages, not PMNs, had the greater capacity for *C. albicans* uptake despite engulfing fungal cells at a lower rate after establishing cell-cell contact. However, when both cell types were present, macrophages and PMNs contributed approximately equally to clearance of *C. albicans*. Our data illustrate that uptake rates are affected not only by the fungal/phagocyte ratio but also by the proportion of the phagocyte subsets encountered. This suggests that the overall phagocytic capacity in single phagocyte assays is not determined simply by the number PMNs and macrophages but is rather a consequence of the percentage of phagocytes contributing to uptake. *In vivo*, the contribution of phagocyte subsets to overall uptake would also be influenced further by factors such as chemokine and cytokine concentrations and spatial considerations such as location of the fungal infiltrate, the numbers of phagocytes, and accessibility to the infection sites. Interestingly, observations from a larval zebrafish model of disseminated candidiasis where phagocytosis of *Candida* cells was imaged *in vivo* showed that macrophage-like cells took up a significantly greater number of *C. albicans* yeast cells than neutrophils did (17).

A number of studies have previously established that *C. albicans* can form filaments inside macrophages and that hyphal formation is essential for *C. albicans* escape and phagocyte killing (8, 30–32). In this study, we examined this phenomenon further and observed that PMNs were significantly more susceptible than macrophages to *C. albicans*-mediated killing when incubated in isolation with *C. albicans*. Indeed, *in vitro*, nearly 50% of macrophages that took up 5 or more fungal cells survived, whereas 100% of PMNs that took up 3 or more fungal cells were killed following continued growth of hyphae inside the host cell. However, when both macrophages and PMNs were studied in coculture, macrophage survival following *C. albicans* phagocytosis was significantly impaired, and the rate of *C. albicans*-mediated killing of macrophages increased to the same level as that of PMNs incubated alone with *C. albicans*.

We showed here that PMNs not only migrated more rapidly than macrophages toward *C. albicans* cells but also engulfed *C. albicans* cells at a significantly higher rate once cell-cell contact was established. The differential activation and expression of PRRs displayed on macrophages and PMNs may contribute to the observed differences in rates of engulfment seen in these myeloid cells in the present study. This is in keeping with the results of a number of studies that have established that immune cell migration and fungal cell recognition is influenced by the relative levels of expression and specificities of PRRs displayed on the immune cell surface (34,35). The observations also mirror findings from our previous work showing that the glycosylation status of the *C. albicans* cell wall and therefore repertoire of PAMPs affects the migration, engulfment, and killing of neutrophils (5) and macrophages (28).

Taken together, the data from the current study suggest that when both macrophages and PMNs are presented to *C. albicans* in a coculture, PMNs mediate the majority of early *C. albicans* yeast cell uptake events due to their ability to migrate more rapidly toward fungal cells and then engulf bound fungal cells at a higher rate than macrophages do. As a consequence, macrophages may encounter a higher proportion of hyphal cells, which they engulf at a lower rate. Both uptake of a higher proportion of hyphal *C. albicans* cells and increased hyphal length at the time of uptake is reportedly associated with host cell death (8, 28, 31, 32), which may explain the increase in macrophage death seen here in the coculture assay compared to that when macrophages were incubated alone with *C. albicans*.

This study highlights the critical role that macrophages and PMNs play in *C. albicans* phagocytosis and demonstrates that major kinetic parameters related to the rate and efficiency of phagocytosis and killing are multifactorial, depending on the presence of multiple cell types and a temporal program of events that ultimately defines the outcome of the predation of fungal cells by different phagocyte cell types. The implications of this study are that extrapolations of complex interactions between pathogen cells and the myeloid cells involved in innate immunity have to be made in the knowledge that cellular interactions are significantly affected by the proximity of other cell types and dynamic changes in the predator/prey cell type ratio.

## MATERIALS AND METHODS

### *C. albicans* strains and growth conditions.

*C. albicans* serotype A strain CAI4 + CIp10 was used for all experiments and was the progenitor of the mutant strains used in our previous studies (28). This strain was obtained from glycerol stocks stored at −80°C and plated on synthetic complete medium lacking uracil (SC−Ura). SC−Ura plates consisted of 6.9 g yeast nitrogen base without amino acids (Formedium, Norfolk, United Kingdom), 1 ml 1 M NaOH (BDH Chemicals, VWR International, Leicestershire, United Kingdom), 10 ml 1% (wt/vol) adenine hemisulfate salt (Sigma, Dorset, United Kingdom), 50 ml 40% d-glucose (Fisher Scientific, Leicestershire, United Kingdom), 50 ml 4% SC−Ura dropout (Formedium, Norfolk, United Kingdom), and 2% (wt/vol) technical agar (Oxoid, Cambridge, United Kingdom) made up to 1,000 ml in distilled H_2_O.

### Preparation of human monocyte-derived macrophages.

Human monocyte-derived macrophages were isolated from the blood of healthy volunteers. Peripheral venous blood was collected in EDTA-coated tubes, pooled, and diluted 1:3 in Hanks’ balanced salt solution (HBSS). Lymphoprep (Axis-Shield, Norway) kits were used to separate peripheral blood mononuclear cells (PBMCs) from whole blood. The PBMC layer was washed and resuspended in DMEM (Lonza, Slough, United Kingdom) supplemented with 200 U/ml penicillin-streptomycin antibiotics (Invitrogen, Paisley, United Kingdom) and 2 mM l-glutamine (Invitrogen, Paisley, United Kingdom). Serum was separated from blood using standard methods and heat inactivated at 56°C for 20 min before use. PBMCs were seeded at 10 × 10^6^ in 2 ml supplemented DMEM containing 10% autologous human serum onto glass-based imaging dishes (PAA Imaging gas chromatography [GC] dishes; The Cell Culture Company, GE Healthcare, France) and incubated at 37°C with 5% CO_2_ for 2 h to allow for monocyte adherence to the dish. After 2 h, the supernatant containing the floating lymphocytes was removed and replaced with 2 ml warm supplemented DMEM containing 10% autologous human serum. Dishes were incubated at 37°C with 5% CO_2_, and medium was changed on days 3 and 6. Cells were used in imaging experiments on day 7. Immediately prior to phagocytosis experiments, supplemented DMEM was replaced with prewarmed supplemented serum-free CO_2_-independent medium (Gibco, Invitrogen, Paisley, United Kingdom) containing 1 µM LysoTracker red DND-99 (Invitrogen, Paisley, United Kingdom) when macrophages were the only immune cell type present or 1 µM LysoTracker blue DND-22 (Invitrogen, Paisley, United Kingdom) when both macrophages and PMNs were present to distinguish between the two immune cell types. LysoTracker red and blue DND are fluorescent dyes that stain acidic compartments in live cells, enabling tracking of these cells during phagocytosis and phagolysosome maturation.

### Preparation of human PMNs.

Human PMNs were isolated from the blood of healthy volunteers and used in phagocytosis assays within 3 h of isolation. Peripheral venous blood was collected in EDTA-coated tubes, pooled, and diluted 1:2 in sterile PBS. Histopaque-1119 (Sigma, Dorset, United Kingdom) was added to a conical tube and carefully overlaid with the same volume of Histopaque-1077 (Sigma, Dorset, United Kingdom) to create a double-density gradient. An equal volume of diluted blood was carefully layered on top of the upper Histopaque gradient, and tubes were centrifuged at 700 × *g* for 40 min at room temperature to allow for separation of PBMCs and PMNs from erythrocytes. Serum and PBMC layers were aspirated and discarded, and the PMN layer was washed once in PBS. For hypotonic lysis of erythrocytes carried over into the PMN fraction, 3 ml of 0.2% NaCl solution was added to cells and mixed gently. After 30 to 45 s, 3 ml of 1.6% NaCl solution was added, mixed gently, and then diluted to 50 ml in PBS before centrifugation at 350 × *g* for 10 min at room temperature. Cells were then washed in PBS and resuspended in prewarmed supplemented serum-free CO_2_-independent medium containing 1 µM LysoTracker red DND‑99 (Invitrogen, Paisley, United Kingdom) and used immediately in phagocytosis assays.

### Preparation of FITC-stained *C. albicans*.

*C. albicans* colonies were grown in SC−Ura medium and incubated overnight at 30°C with 200-rpm shaking. Live *C. albicans* cells were stained for 10 min at room temperature in the dark with 1 mg/ml fluorescein isothiocyanate (FITC) (Sigma, Dorset, United Kingdom) in 0.05 M carbonate-bicarbonate buffer (pH 9.6) (BDH Chemicals, VWR International, Leicestershire, United Kingdom). Following incubation, cells were washed three times in 1× PBS to remove any residual FITC and finally resuspended in 1× PBS.

### Live-cell video microscopy phagocytosis assays.

Phagocytosis assays were performed using our standard protocol, with modifications (8, 28). Samples of live FITC-stained wild-type *C. albicans* (CA14-CIp10) cells were added to 1 × 10^6^ LysoTracker red DND-99-stained human monocyte-derived macrophages or 1 × 10^6^ LysoTracker red DND-99-stained human PMNs in glass-based imaging dishes at a multiplicity of infection (MOI) of either 1 or 3. For phagocytosis assays where both macrophages and PMNs were present at a 1:1 ratio, live, FITC-stained wild-type *C. albicans* (CA14-CIp10) cells were added to glass-based imaging dishes containing 5 × 10^5^ macrophages stained with 1 µM LysoTracker blue DND-22, and 5 × 10^5^ PMNs stained with 1 µM LysoTracker red DND-99 at an MOI of 3 (ratio of *C. albicans* cells to total phagocytes, 3:1). In the coculture experiments, macrophages and PMNs were not always isolated from the same donors. Video microscopy was performed using a DeltaVision Core microscope (Applied Precision, WA, USA) in a 37°C chamber, and images were captured at 1-min intervals over a 6-h period using an EMCCD camera. At least three independent experiments were performed for both macrophages and PMNs incubated alone with *C. albicans*, and at least 3 videos were analyzed from each experiment using softWoRx Explorer image analysis software (Applied Precision). The same setup was used for the coculture assays where both macrophages and PMNs were present. One hundred phagocytes were selected at random from each experiment and analyzed individually at 1-min intervals over a 6-h period. Measurements taken included *C. albicans* uptake, defined as the number of *C. albicans* cells taken up by an individual phagocyte over the 6-h period, the time point at which cell-cell contact was established, and the time point at which a *C. albicans* cell was fully engulfed; these measurements were used to determine the rate of *C. albicans* engulfment, defined as the time taken from establishment of cell-cell contact to complete ingestion of a *C. albicans* cell (a fungal cell was considered to have been fully ingested when its FITC fluorescent signal was lost, indicating that the fungal cell was now inside the phagocyte and not merely bound to the phagocyte cell surface). The viability of the phagocytes over the 6-h period was defined as the percentage of macrophages or PMNs from the total macrophage or PMN population, respectively, that had been killed by specific time points. In the coculture assays, macrophages and PMNs were also analyzed separately in this manner, so the percentage of macrophages killed by certain time points in the coculture assays represents the percentage of macrophages out of the total macrophage population sampled that had been killed. Velocity 6.2 imaging analysis software was used to track phagocyte migration at 1-min intervals throughout the 6-h phagocytosis assay. The software enabled high-throughput analysis of phagocyte migration, providing detailed information on the distances traveled, directionality, and velocity of hundreds of individual phagocytes. Data were subsequently displayed in tracking diagrams and used to calculate the mean track velocity and track lengths of phagocytes cultured with *C. albicans* (28).

Means values and standard deviations were calculated. Unpaired, two-tailed *t* tests and two-way analysis of variance (ANOVA) followed by Bonferroni multiple-comparison tests were used to determine statistical significance.

## SUPPLEMENTAL MATERIAL

Video S1 Macrophages incubated alone with *C. albicans* for 6 h (macrophages are stained red, and *C. albicans* cells are stained green). Download Video S1, WMV file, 3.4 MB

Video S2 Neutrophils incubated alone with *C. albicans* for 6 h (neutrophils are stained red, and *C. albicans* cells are stained green). Download Video S2, MPG file, 9 MB

Video S3 Neutrophils incubated alone with *C. albicans* over 3 h taken with a spinning-disk microscope (neutrophils are stained red, and *C. albicans* cells are stained green). Download Video S3, WMV file, 4.2 MB

Video S4 Three-dimensional video demonstrating *C. albicans* engulfment by neutrophils using a spinning-disk microscope (neutrophils are stained red, and *C. albicans* cells are stained green). Download Video S4, WMV file, 18.5 MB

Video S5 Macrophages and neutrophils incubated together with *C. albicans* in the coculture assay for 6 h (macrophages are stained blue, neutrophils are stained red, and *C. albicans* cells are stained green). Download Video S5, WMV file, 2.6 MB

Video S6 *C. albicans* hyphal extension during uptake by a macrophage (the macrophage is stained blue, and the *C. albicans* cell is stained green). Download Video S6, WMV file, 3.2 MB

## References

[1] Spellberg B 2011 Vaccines for invasive fungal infections. F1000 Med. Rep. 3:13. 10.3410/M3-13 21876719 PMC3155210

[2] Zaoutis TE Argon J Chu J Berlin JA Walsh TJ Feudtner C 2005 The epidemiology and attributable outcomes of candidemia in adults and children hospitalized in the United States: a propensity analysis. Clin. Infect. Dis. 41:1232–1239 16206095 10.1086/496922

[3] Liu Y Filler SG 2011 *Candida albicans* Als3, a multifunctional adhesin and invasin. Eukaryot. Cell 10:168–173 21115738 10.1128/EC.00279-10 PMC3067396

[4] Fu Y Ibrahim AS Sheppard DC Chen YC French SW Cutler JE Filler SG Edwards JE Jr 2002 *Candida albicans* Als1p: an adhesin that is a downstream effector of the EFG1 filamentation pathway. Mol. Microbiol. 44:61–72 11967069 10.1046/j.1365-2958.2002.02873.x

[5] Sheth CC Hall R Lewis L Brown AJP Odds FC Erwig LP Gow NAR. 2011 Glycosylation status of the *C. albicans* cell wall affects the efficiency of neutrophil phagocytosis and killing but not cytokine signaling. Med. Mycol. 49:513–524 21254968 10.3109/13693786.2010.551425 PMC3119872

[6] Netea MG Brown GD Kullberg BJ Gow NAR 2008 An integrated model of the recognition of *Candida albicans* by the innate immune system. Nat. Rev. Microbiol. 6:67–78 18079743 10.1038/nrmicro1815

[7] Miramón P Dunker C Windecker H Bohovych IM Brown AJP Kurzai O Hube B 2012 Cellular responses of *Candida albicans* to phagocytosis and the extracellular activities of neutrophils are critical to counteract carbohydrate starvation, oxidative and nitrosative stress. PLoS One 7:e52850. 10.1371/journal.pone.0052850 23285201 PMC3528649

[8] McKenzie CGJ Koser U Lewis LE Bain JM Mora-Montes HM Barker RN Gow NAR Erwig LP 2010 Contribution of *Candida albicans* cell wall components to recognition by and escape from murine macrophages. Infect. Immun. 78:1650–1658 20123707 10.1128/IAI.00001-10 PMC2849426

[9] Cheng SC Joosten LAB Kullberg BJ Netea MG 2012 Interplay between *Candida albicans* and the mammalian innate host defense. Infect. Immun. 80:1304–1313 22252867 10.1128/IAI.06146-11 PMC3318407

[10] Traynor TR Huffnagle GB 2001 Role of chemokines in fungal infections. Med. Mycol. 39:41–50 11270407 10.1080/mmy.39.1.41.50

[11] Diamond RD 1993 Interactions of phagocytic cells with *Candida* and other opportunistic fungi. Arch. Med. Res. 24:361–369 8118160

[12] Maertens J Vrebos M Boogaerts M 2001 Assessing risk factors for systemic fungal infections. Eur. J. Cancer Care (Engl.) 10:56–62 11827268 10.1046/j.1365-2354.2001.00241.x

[13] Martino P Girmenia C Venditti M Micozzi A Santilli S Burgio VL Mandelli F 1989 *Candida* colonization and systemic infection in neutropenic patients. A retrospective study. Cancer 64:2030–2034 2804891 10.1002/1097-0142(19891115)64:10<2030::aid-cncr2820641011>3.0.co;2-2

[14] Lehrer RI Cline MJ 1969 Leukocyte myeloperoxidase deficiency and disseminated candidiasis: the role of myeloperoxidase in resistance to *Candida* infection. J. Clin. Invest. 48:1478–1488 5796360 10.1172/JCI106114 PMC322375

[15] El-Maallem H Fletcher J 1977 Impaired neutrophil function and myeloperoxidase deficiency in myeloid metaplasia. Br. J. Haematol. 37:323–329 203312 10.1111/j.1365-2141.1977.tb01002.x

[16] Qian Q Jutila MA Van Rooijen N Cutler JE 1994 Elimination of mouse splenic macrophages correlates with increased susceptibility to experimental disseminated candidiasis. J. Immunol. 152:5000–5008 8176217

[17] Romani L 2011 Immunity to fungal infections. Nat. Rev. Immunol. 11:275–288 21394104 10.1038/nri2939

[18] Medzhitov R 2007 Recognition of microorganisms and activation of the immune response. Nature 449:819–826 17943118 10.1038/nature06246

[19] Mora-Montes HM Netea MG Ferwerda G Lenardon MD Brown GD Mistry AR Kullberg BJ O’Callaghan CA Sheth CC Odds FC Brown AJP Munro CA Gow NAR 2011 Recognition and blocking of innate immunity cells by *Candida albicans* chitin. Infect. Immun. 79:1961–1970 21357722 10.1128/IAI.01282-10 PMC3088140

[20] Seider K Heyken A Lüttich A Miramón P Hube B 2010 Interaction of pathogenic yeasts with phagocytes: survival, persistence and escape. Curr. Opin. Microbiol. 13:392–400 20627672 10.1016/j.mib.2010.05.001

[21] Amulic B Cazalet C Hayes GL Metzler KD Zychlinsky A 2012 Neutrophil function: from mechanisms to disease. Annu. Rev. Immunol. 30:459–489 22224774 10.1146/annurev-immunol-020711-074942

[22] Brothers KM Newman ZR Wheeler RT 2011 Live imaging of disseminated candidiasis in zebrafish reveals role of phagocyte oxidase in limiting filamentous growth. Eukaryot. Cell 10:932–944 21551247 10.1128/EC.05005-11 PMC3147414

[23] Brown GD 2011 Innate antifungal immunity: the key role of phagocytes. Annu. Rev. Immunol. 29:1–21 20936972 10.1146/annurev-immunol-030409-101229 PMC3434799

[24] Urban CF Reichard U Brinkmann V Zychlinsky A 2006 Neutrophil extracellular traps capture and kill *Candida albicans* yeast and hyphal forms. Cell. Microbiol. 8:668–676 16548892 10.1111/j.1462-5822.2005.00659.x

[25] Vijayan D Radford KJ Beckhouse AG Ashman RB Wells CA 2012 Mincle polarizes human monocyte and neutrophil responses to *Candida albicans*. Immunol. Cell Biol. 90:889–895 22641025 10.1038/icb.2012.24

[26] Keppler-Ross S Douglas L Konopka JB Dean N 2010 Recognition of yeast by murine macrophages requires mannan but not glucan. Eukaryot. Cell 9:1776–1787 20833894 10.1128/EC.00156-10 PMC2976302

[27] Gow NAR Van De Veerdonk FL Brown AJP Netea MG 2012 *Candida albicans* morphogenesis and host defence: discriminating invasion from colonization. Nat. Rev. Microbiol. 10:112–122 22158429 10.1038/nrmicro2711 PMC3624162

[28] Lewis LE Bain JM Lowes C Gillespie C Rudkin FM Gow NAR Erwig LP 2012 Stage specific assessment of *Candida albicans* phagocytosis by macrophages identifies cell wall composition and morphogenesis as key determinants. PLoS Pathog. 8:e1002578. 10.1371/journal.ppat.1002578 22438806 PMC3305454

[29] Bain JM Lewis LE Okai B Quinn J Gow NAR Erwig LP 2012 Non-lytic expulsion/exocytosis of *Candida albicans* from macrophages. Fungal Genet. Biol. 49:677–678 22326419 10.1016/j.fgb.2012.01.008 PMC3430864

[30] Lewis LE Bain JM Lowes C Gow NAR Erwig LP 2012 *Candida albicans* infection inhibits macrophage cell division and proliferation. Fungal Genet. Biol. 49:679–680 22634272 10.1016/j.fgb.2012.05.007 PMC3430961

[31] Ghosh S Navarathna DHMLP Roberts DD Cooper JT Atkin AL Petro TM Nickerson KW 2009 Arginine-induced germ tube formation in *Candida albicans* is essential for escape from murine macrophage line RAW 264.7. Infect. Immun. 77:1596–1605 19188358 10.1128/IAI.01452-08 PMC2663133

[32] Lorenz MC Bender JA Fink GR 2004 Transcriptional response of *Candida albicans* upon internalization by macrophages. Eukaryot. Cell 3:1076–1087 15470236 10.1128/EC.3.5.1076-1087.2004 PMC522606

[33] Ermert D Niemiec MJ Röhm M Glenthøj A Borregaard N Urban CF. 2013 *Candida albicans* escapes from mouse neutrophils. J. Leukoc. Biol. 94:223-236 23650619 10.1189/jlb.0213063

[34] Bain JM Lewis LE Erwig LP. 2012 Differential expression of macrophage and neutrophil phagocytic receptors recognising fungal pathogens in mouse and human. Immunol. Cell Biol. 90:837-838 22801576 10.1038/icb.2012.38

[35] Klaas M Oetke C Lewis LE Erwig LP Heikema AP Easton A Willison HJ Crocker PR. 2012 Sialoadhesin promotes rapid proinflammatory and type I IFN responses to a sialylated pathogen, *Campylobacter jejuni*. J. Immunol. 189:2414-2422 22851711 10.4049/jimmunol.1200776 PMC3442253

